# Household Responses to Pandemic (H1N1) 2009–related School Closures, Perth, Western Australia

**DOI:** 10.3201/eid1602.091372

**Published:** 2010-02

**Authors:** Paul V. Effler, Dale Carcione, Carolien Giele, Gary K. Dowse, Leigh Goggin, Donna B. Mak

**Affiliations:** Associate Editor, Emerging Infectious Diseases journal, Centers for Disease Control and Prevention, Atlanta, Georgia, USA (P.V. Effler); Department of Health, Perth, Western Australia, Australia (P.V. Effler, D. Carcione, C. Giele, G.K. Dowse, L. Goggin, D.B. Mak)

**Keywords:** Influenza A virus, schools, pandemic (H1N1) 2009, H1N1 subtype, pandemic, transmission, pandemic response, influenza, research, expedited

## Abstract

School closure is often purported to reduce influenza transmission, but little is known about its effect on families. We surveyed families affected by pandemic (H1N1) 2009–related school closures in Perth, Western Australia, Australia. Surveys were returned for 233 (58%) of 402 students. School closure was deemed appropriate by 110 parents (47%); however, 91 (45%) parents of 202 asymptomatic students reported taking >1 day off work to care for their child, and 71 (35%) had to make childcare arrangements because of the class closures. During the week, 172 (74%) students participated in activities outside the home on >1 occasion, resulting in an average of 3.7 out-of-home activities for each student. In our survey, activities outside the home were commonly reported by students affected by school closure, the effect on families was substantial, and parental opinion regarding school closures as a means to mitigate the outbreak of pandemic (H1N1) 2009 was divided.

## Abstract 2

Results from closures will determine the appropriateness and efficacy of this mitigation measure.

On Friday, June 5, 2009, the Western Australia Department of Health received its third notification of confirmed infection with influenza A pandemic (H1N1) 2009. The patient was an elementary school student from Perth, Western Australia, Australia, who had recently returned from a sporting club excursion to Victoria, another Australian state, which had already experienced >600 cumulative confirmed cases of pandemic influenza (*1*).

Over the next 3 days (June 6–8), vigorous contact tracing and testing confirmed 11 more pandemic (H1N1) 2009 infections among schoolchildren; all had either visited Victoria or were close contacts of confirmed case-patients who had traveled to Victoria. These 11 children attended 3 schools located within 2 km of each other in a socioeconomically advantaged area of Western Australia’s capital, Perth, which has a population of 1.7 million (*2*). On Sunday, June 7, in accordance with Australian public health practice at the time, the Department of Health advised the 3 schools to cancel classes for the coming week. School A, a public school, closed entirely; schools B and C, both private, cancelled classes for grade 5 and grades 5–7, respectively. The grades closed at schools B and C were those in which at least 1 student was confirmed as having pandemic (H1N1) 2009 virus infection.

School closure (i.e., either closure of school or dismissal of classes) is a nonpharmaceutical intervention often recommended for mitigating virus transmission during an influenza pandemic (*3*,*4*). However, little is known about the effect of school closures on students and families. We describe the activities of students affected by school closure, the effect of school closure on families, and parental opinions regarding school closures implemented in response to influenza A pandemic (H1N1) 2009.

## Methods

Parents of all students excluded from attendance at schools A, B, and C were surveyed to ascertain the age of their child; the onset of illness, if any, in their child during the school closure period; the need for special childcare arrangements due to the closure; whether the child went out of the home during the school closure period; and parental perspectives on the consequences and appropriateness of the school closure. Parents were asked to complete a written questionnaire for their child; no personal identifiers were obtained. Surveys were distributed by schools on June 22, 2009 (10 days after school closure ended), and collected on July 3, 2009.

A case-patient was defined as a student with PCR results positive for influenza A pandemic (H1N1) 2009 virus. A contact was defined as a student who had been in a classroom with a case-patient for >4 hours or who had had another period of close physical proximity (e.g., sitting within 1 m of the case-patient for at least 15 min) during the case-patient’s infectious period (i.e., 1 day before until 7 days after symptom onset). Other students affected by the closure but who did not meet case-patient or contact criteria were defined as school peers.

Influenza-like illness (ILI) was defined as an illness with fever and cough and/or sore throat. Upper respiratory infection (URI) was defined as an illness not meeting ILI criteria but exhibiting >1 of the following signs or symptoms: sore throat, cough or runny nose. Asymptomatic students were defined as contacts and peers in whom ILI or URI did not develop during the period of school closure. Ill students were defined as case-patients, contacts, and peers who developed ILI or URI during the school closure period.

**Figure 1:**
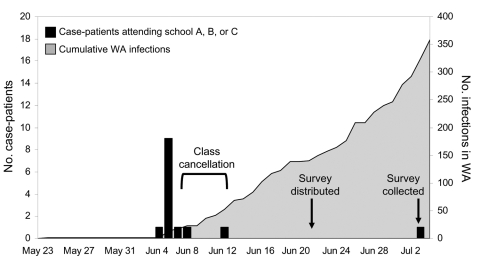
Confirmed pandemic (H1N1) 2009 influenza infections in Western Australia (WA), by onset date, May 23–July 4, 2009.

The school closure period was defined as the interval from the first day classes were cancelled to the first day classes resumed (i.e., June 8–14, 2009) (Figure 1). Special childcare arrangements were defined as childcare activities other than the students caring for themselves at home or care provided by an adult member of the household. To separate the effects of the school closure by itself from the consequences of caring for a symptomatic child, the analysis of parental time off work and special childcare arrangements was limited to parents of asymptomatic students.

Frequencies, means, proportions, and Kruskal-Wallis tests of significance were calculated by using Epi Info 2000 (Centers for Disease Control and Prevention, Atlanta, GA, USA; www.cdc.gov/epiinfo/epiinfo.htm). Pearson χ^2^ statistics were obtained by using SPSS version 17.0 software (SPPS Inc., Chicago, IL, USA).

## Results

Surveys were returned for 233 (58%) of the 402 students affected by school closure; 49%, 59%, and 67% of surveys from schools A, B, and C, respectively, were returned. The median age of the students was 11 years (range 5–13 years).

**Figure 2:**
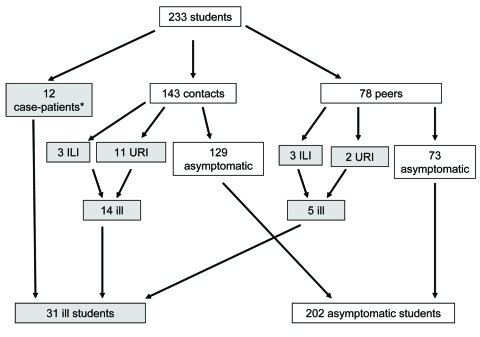
Distribution of student respondents by case or exposure classification and history of illness during a pandemic (H1N1) 2009–related school closure, Perth, Western Australia, June 8–14, 2009. URI, upper respiratory infection; ILI, influenza-like illness.

Of the 233 responses, 12 (5%) were from households with case-patients in the initial cluster of pandemic (H1N1) 2009 infections that led to the recommendation for school closure; 143 (61%) of the responses were from households with contacts of case-patients, and 78 (34%) were from households with peers (Figure 2).

Of 221 contacts and peers, 19 (9%) reported onset of respiratory symptoms during the week of school closure; 14 were contacts and 5 were peers. Illness in 6 of the symptomatic students (3 contacts and 3 peers) met the criteria for ILI; the remaining illnesses were URIs (Table 1).

**Table 1:** Symptoms reported by contacts and peers who became ill during pandemic (H1N1) 2009–related school closures, Perth, Western Australia, Australia, June 8–14, 2009

Sign or symptom	No. (%) persons with ILI,* n = 6	No. (%) persons with URI,† n = 13	Total no. (%) persons, n = 19
Fever	6 (100)	1 (8)	7 (37)
Sore throat	6 (100)	6 (46)	12 (63)
Headache	5 (83)	4 (31)	9 (47)
Cough	4 (67)	4 (31)	8 (42)
Runny nose	4 (67)	7 (54)	11 (58)
Sneezing	3 (50)	2 (15)	5 (26)
Chills	2 (33)	0	2 (11)
Fatigue	1 (17)	1 (8)	2 (11)
Muscle aches	0	2 (15)	2 (11)
Diarrhea	0	1 (8)	1 (5)
Vomiting	0	2 (15)	2 (11)
Other	0	4 (31)	4 (21)

*ILI, influenza-like illness; defined as a history of fever with cough, sore throat, or both. †URI, upper respiratory infection; defined as an illness not meeting ILI criteria but with >1 of the following: sore throat, cough, or runny nose.

A total of 172 students (74%) reported going outside the home during the school closure period. In aggregate, students reported a total of 860 out-of-home activities during the school closure, resulting in a mean of 3.7 activities per student/week (a mean of 2.2 out-of-home activities per student during the 5 weekdays and 1.5 on the weekend). The number of out-of-home activities reported by individual students ranged from 0 to 24 (median 3 activities). Common reasons for going outside the home included sporting events, outdoor recreation, shopping, and parties (Table 2).

**Table 2:** Number and type of out-of-home activities reported by 233 students during pandemic (H1N1) 2009–related school closures, Perth, Western Australia, Australia, June 8–14, 2009

Activity type	No. students reporting activity		
	Monday–Friday (Jun 8–12)	Saturday–Sunday (Jun 13–14)	Total
Going to a sporting event	86	128	214
Going to a park or beach	144	52	196
Going to a grocery store	68	35	103
Going to a shopping mall	63	28	91
Participating in an unspecified event or activity	58	17	75
Attending a party	28	37	65
Attending a music or art lesson	19	8	27
Going to a restaurant	13	14	27
Going to a movie	16	5	21
Attending a religious service	1	16	17
Attending a sleepover	10	6	16
Going to a tutoring lesson	6	2	8
Total	512	348	860

There was a significant difference in the proportion of case-patients (42%), contacts (66%), and peers (92%) who reported going out of the home >1 time during the closure period (Pearson χ^2^ = 24.4, df = 2, p<0.0001). The mean number of times students reported going out of the home was also associated with whether the student was a case-patient, contact, or peer; case-patients reported an average of 0.8 out-of-home activities per student per week compared with contacts and peers, who reported a mean of 2.9 and 5.6 activities per student per week, respectively (Kruskal-Wallis H = 35.1, df = 2, p<0.0001).

A total of 91 (45%) parents of 202 asymptomatic students reported taking >1 day (range 1–5 days; median 3 days) off work to care for their child during the closure period. Seventy-one parents (35%) of asymptomatic students reported having to make special childcare arrangements as a result of the school closures. The median number of days that special childcare arrangements were required was 2 (range 1–5 days). Twenty students (10%) cared for themselves at home for at least a portion of the closure period. Of the 202 asymptomatic students and of 31 ill students, 38 (19%) and 2 (6%), respectively, were cared for in a setting with children other than their siblings.

Of the 233 parents who returned the survey, 110 (47%) thought the school closure was appropriate, 76 (33%) thought it was inappropriate, and 47 (20%) were unsure. The percentage of parents who indicated that the closure was appropriate was highest among the parents of case-patients (92%), followed by the parents of contacts (48%), and peers (39%). Of those parents who thought school closure was appropriate, the main reason cited was to “protect the community”; of parents who thought the school closures were not appropriate, the main reason cited was “swine flu illness is mild” (Table 3).

**Table 3:** Household responses to survey regarding pandemic (H1N1) 2009–related school closures, Perth, Western Australia, 2009, June 8–14, 2009

Survey question, response	No. (%) responses
Was school closure appropriate, and why or why not?*	
Yes	110 (47)
To protect the community	84 (76)
To protect other students at the school	72 (66)
To protect my child and family	68 (62)
Swine flu illness is serious	31 (28)
No	76 (33)
Swine flu illness is mild	54 (71)
Cannot stop flu spread	50 (66)
Lost income due to missed work	18 (24)
Too difficult to make childcare arrangements	14 (18)
Not sure	47 (20)
What was the level of disruption to family routines caused by the closure?	
Severe	32 (14)
Moderate	95 (41)
Minimal	87 (37)
None	19 (8)
What level of anxiety did the closure create in your child?	
Severe	3 (1)
Moderate	20 (9)
Minimal	101 (43)
None	109 (47)
What could have helped you be bettered prepared for the school closure?*	
Nothing, we were well prepared	104 (45)
More time between notification and closing	47 (20)
Assistance with emergency childcare arrangements	45 (19)
Better understanding of potential length of closure at outset	26 (11)

*Reasons for the individual responses were not mutually exclusive.

Parental opinion about the appropriateness of the school closure was significantly correlated with student participation in activities outside the home. Students of parents who thought the school closure was not appropriate reported a mean of 4.7 out-of-home activities, compared with a mean of 4.3 activities for students of parents who were unsure and 2.8 for students of parents who thought the closure was appropriate (Kruskal-Wallis H = 14.9, df = 2, p = 0.0006). This pattern persisted when the analysis was restricted to the 202 students who were asymptomatic (Kruskal-Wallis H = 7.1, df = 2, p = 0.03).

Ninety percent of parents reported that the school closure caused minimal or no anxiety for their child, but 55% reported that school closure caused moderate or severe disruption to family routines. Forty-five percent indicated that they were well prepared for school closure (Table 3).

## Discussion

This report describes the activities of students affected by school closure in response to the outbreak of pandemic (H1N1) 2009 and the effect of the closures on families. It is important to place these findings in context to fully understand their importance. The school closures in Perth occurred at a time when experience with pandemic (H1N1) 2009 was limited but transmission of the virus was accelerating across Australia. By June 8, 2009, >1,600 confirmed cases of pandemic (H1N1) 2009 illness had been reported nationally, and 10 fatal infections had received extensive media coverage. Thermal scanners were operational at Australia’s international airports, and cruise ships with suspected case-patients had recently been quarantined at sea (*5*,*6*). During this phase of the response, case-patients and contacts were routinely provided with oseltamivir and instructed to voluntarily isolate or quarantine themselves at home. In addition, as a precaution, all school-aged children who traveled to areas with high rates of pandemic (H1N1) 2009, specifically the United States, Canada, Mexico, and Victoria, Australia, were being asked to remain in home quarantine for 7 days after return (*7*,*8*). The school closures in Western Australia were covered widely by the local media, and the message conveyed was that the school children were to be placed under “home quarantine” (*9*–*11*).

In this setting, less than half of parents (47%) felt that closing schools was an appropriate response to identification of pandemic influenza cases among students. Most parents who felt the closure was inappropriate indicated that their opinion was based on impressions about the disease itself (i.e., that it was generally mild and/or that transmission was not likely to be stopped by closing schools). Personal inconvenience, such as lost work or wages and childcare issues, seemed to be less important.

The effect of the closure on families was substantial. Almost half of parents reported missing work because of the school closure, and a third had to make special childcare arrangements. These special arrangements resulted in >1 of 6 students being cared for with children other than their siblings.

During the school closure, respiratory illness developed in 14 (10%) of 143 contacts and 5 (6%) of 78 peers; 6 of the 19 illnesses met the case definition for ILI, but the remaining URIs were mostly afebrile. Anecdotal reports from Mexico and elsewhere suggest that 30% of illnesses caused by pandemic (H1N1) 2009 may lack fever, and volunteer challenge studies with other influenza subtype H1N1 viruses have found the proportion of afebrile infections to be even higher (63%) (*12*,*13*). Clinical specimens were not available from ill students reporting ILI or URI in this assessment, so we are not able to establish the true incidence of influenza in our cohort.

Students commonly reported participating in activities outside the home during the school closure. Almost three quarters of all students left home at least 1 time during the 7-day reporting period.

This survey provides quantitative data on the frequency of students’ out-of-home activities during school closures undertaken to control an outbreak. Understanding what children do when schools are closed is crucial to predicting the health effects of illness-related school closures (*14*,*15*). The 233 students in our survey reported, in aggregate, >850 out-of-home activities over 7 days. This may actually be an underestimate as it represents only what was later recalled by parents (*16*).

Studies attempting to simulate the impact of nonpharmaceutical interventions on pandemic transmission have found that the anticipated benefits of school closures may be substantially undermined if children are not sufficiently isolated (*14*,*17*). Simulation models that include school closures are sensitive to assumptions regarding the level of nonschool contacts that occur (*18*,*19*). The large potential reductions in disease transmission predicted by some modeling studies are often based on the assumption that students stay at home during school closures or that compliance with social distancing recommendations is high (e.g., 90%) (*20*,*21*). Our data suggest the assumption that children will be kept at home may not be realistic for the current outbreak of pandemic (H1N1) 2009. Of the 3 student cohorts we surveyed (case-patients, contacts, and peers), peers are likely to be representative of the majority of children affected during a large-scale school closure scenario; we observed that peers went out of the home nearly once a day, on average, during the survey period. How this behavior might have changed if the exclusion period had been longer is unknown.

It is likely that there is wide variability in terms of the risk for influenza transmission across the spectrum of outside-the-home activities the students engaged in (i.e., differences in duration and proximity of exposure to other persons). In our setting, sporting events (team practices and games) were the most common activity reported and have been recognized for their potential association with the spread of influenza (*3*). In contrast, excursions to a beach or park were also common, but these activities are probably less likely to result in disease spread. Parties were less frequent but not uncommon, and social events of this type have recently been implicated in transmission of pandemic (H1N1) 2009 in Europe (*22*). More work is needed to clarify the likelihood of influenza transmission associated with different activities so recommendations on restricting students’ public contact during school closures might be targeted at those interactions that pose substantial risk.

Another key finding of this assessment is that parental opinions about the appropriateness of school closure were correlated with whether the student participated in activities outside the home. If high rates of compliance with student-centered, nonpharmaceutical interventions are to be obtained, public health officials must communicate to parents why the intervention is warranted and explain the anticipated benefits to the community. It is critical, however, that health officials do “not overstate the level of confidence or certainty in the effectiveness of these measures” (*18*).

It seems reasonable to assume that behaviors reported by students during school closures would be highly influenced by the socioeconomic status of the population studied. We note, however, that our results parallel findings from school closures in rural North Carolina, USA, that were implemented in response to a seasonal influenza outbreak. Despite recommendations to avoid large gatherings, most (89%) of the North Carolina students visited at least 1 public location during the 10-day closure (*17*). The general consistency observed in terms of participation in >1 out-of-home activities across the North Carolina and Western Australia settings is noteworthy because the 2 communities seem to be quite distinct with regard to socioeconomic status. In our Western Australia study, the students attended a mix of private and public schools in a relatively urbanized, affluent community; in the North Carolina study, 41% of the participating households received free or reduced-cost lunches through a national school lunch program (an indicator of lower socioeconomic status). Marked differences between the Western Australia and North Carolina assessments were noted, however; for example, in North Carolina, >91% of households considered the school closures appropriate, and no adults reported missing work as a result of the closures (*17*). Additional studies among a wide array of communities will be needed to better elucidate the relationship between socioeconomic status and the effect of school closures on households and student behavior.

This evaluation was performed as a field assessment of an interim, evolving public health response and has several major limitations. First, data were obtained by using a retrospective self-administered questionnaire. Although the response rate (58%) was acceptable, the accuracy of recall may have declined between the end of the closure and the time that the questionnaire was distributed.

Second, we did not collect data on students who were not subject to school closures, so we cannot determine the extent to which student participation in activities outside the home reported by our cohort may have been influenced by the school closure itself (i.e., compensatory behaviors). It is also possible that the number of out-of-home activities per student represents a substantial reduction from “normal” behavior, and thus the school closures could be considered a “qualified success” in terms of reducing student contacts. In addition, it is likely that the overall number of contacts among children and the density of children during the school day exceed the number and density that occur outside the school during a school closure. Whether these plausible, but as-yet-unquantified, relative reductions in student contacts would be sufficient to substantially reduce influenza transmission during a pandemic remains undetermined.

Third, we caution that the responses obtained from Western Australia may not be generalizable to other communities and cultures or to another pandemic with a different disease severity profile. In addition, our findings regarding out-of-school activities may not be applicable to situations in which a total school closure is accompanied by cancellation of all extracurricular school activities. In this assessment, school A closed entirely but schools B and C underwent only partial closure; because schools B and C closed only specific grades, their extracurricular activities were not cancelled and remained open to children in nonaffected grades.

Fourth, our questionnaire did not directly assess parents’ knowledge about why classes were cancelled and what they understood about limiting their child’s activities outside the home. Without this information, it is not possible to determine whether the degree of participation in out-of-home behavior was due to noncompliance or lack of communication.

On the basis of our experience, there are several recommendations future investigators may wish to consider when attempting to assess the effect of illness-related school closures conducted as a disease control measure. These recommendations include 1) directly assessing parental understanding of the recommendations to limit student activities outside the home, 2) concurrently determining the level of out-of-home activities for students at comparable schools not subject to closure, 3) specifically asking about healthcare-seeking behaviors during the school closure, and 4) inquiring about the extent to which any reported outside-of-home behaviors were undertaken as school-affiliated extracurricular activities.

In summary, this study contributes to the growing body of knowledge on student behavior during school closures and the effect of such closures on households. The results of our assessment may be helpful to public health and education officials considering school closure as a means to control an influenza outbreak. In addition, these data might help inform assumptions underpinning studies that estimate the effect of nonpharmaceutical interventions. The general paucity of data on student activities during school closures, however, remains a major barrier to understanding the potential effect of closures as a disease mitigation measure and underscores the need for further research (*18**,**23**–**25*).
